# Attention-Based Quantile Regression for RUL Uncertainty Prediction

**DOI:** 10.3390/s26154748

**Published:** 2026-07-26

**Authors:** Lin Huang, Xianjun Hu, Li Gong, Yajie Liu, Songlin Yang

**Affiliations:** Naval University of Engineering, Wuhan 430033, China

**Keywords:** attention mechanism, quantile regression, RUL, uncertainty prediction

## Abstract

Remaining Useful Life prediction is a core challenge in the field of Prognostics and Health Management. Traditional point prediction methods only provide a single estimate and cannot quantify prediction uncertainty, limiting their application in critical decision-making. This paper proposes a probabilistic prediction model integrating an LSTM–attention mechanism and quantile regression, aiming to achieve interval prediction and uncertainty quantification for RUL. The model employs a bidirectional LSTM network to capture temporal dependencies, focuses on key degradation features through a six-head self-attention mechanism, and outputs predictions for three quantiles (10%, 50%, 90%) simultaneously based on the quantile regression framework, constructing an 80% confidence interval with clear physical meaning. Experimental validation on a public dataset shows that the proposed model performs excellently in both point prediction accuracy and interval prediction quality: RMSE reaches 11.245, NASA Score is 166.414, while the interval coverage remains at 86.7%, and the interval width is 30.398. Ablation experiments further confirm the importance of each component, where removing the attention mechanism causes a 24.4% increase in RMSE, and removing the LSTM module worsens the NASA Score by 164.8%. The research results provide an effective solution for probabilistic remaining life prediction of complex equipment.

## 1. Introduction

In the field of health management for modern industrial systems and complex equipment, accurately predicting the Remaining Useful Life is of great significance for achieving predictive maintenance, reducing operational costs, and improving system reliability. In real industrial or other operating environments, data collected by sensors are often affected by various factors such as noise interference, operational condition changes, and equipment degradation, inevitably introducing a certain degree of uncertainty into the RUL prediction results [1]. In safety-critical domains such as aerospace, energy equipment, military, and medical instruments, understanding prediction uncertainty is even more important than obtaining a single prediction value, as it directly relates to risk assessment and decision-making [2]. Traditional point prediction methods, while providing a single optimal estimate, cannot reflect the inherent uncertainty in the prediction results, which poses limitations in practical engineering decisions. In contrast, probabilistic prediction methods provide a more comprehensive description of the prediction result’s credibility by offering probability distributions or confidence intervals for the predicted values. These methods can not only give the most likely prediction but also quantify the prediction uncertainty, providing richer information for decision-makers [3].

Quantile regression, as an important probabilistic prediction technique, can construct complete prediction intervals by simultaneously estimating predicted values at multiple quantile points. Compared with traditional least-squares regression, quantile regression does not require prior assumptions about the error distribution, is more robust to outliers, and is particularly suitable for processing time-series data with heteroscedasticity [4]. In recent years, with the development of deep learning technology, combining quantile regression with deep neural networks has provided new solutions for probabilistic prediction of complex time-series data [5].

This paper uses the NASA C-MAPSS dataset as an experimental platform, focusing on uncertainty prediction methods for time-series data based on deep learning. We propose a hybrid architecture integrating LSTM networks, attention mechanisms, and quantile regression, aiming to achieve accurate point prediction and reliable interval estimation. This method not only focuses on prediction accuracy metrics like RMSE but also pays more attention to uncertainty quantification metrics such as prediction interval coverage and width. Through systematic experimental validation, we explore the impact of different network structures, loss functions, and training strategies on the effectiveness of uncertainty prediction, providing new technical ideas and implementation schemes for probabilistic RUL prediction of complex systems or equipment. The main contributions of this paper can be summarized as the following four aspects:

1. We proposed a novel hybrid deep learning architecture: We designed an end-to-end probabilistic prediction model combining the temporal modeling capability of LSTM, the feature selection capability of the attention mechanism, and the uncertainty quantification capability of quantile regression, effectively improving the performance of uncertainty prediction for time-series data.
2. We developed a multi-objective optimization strategy: We designed a loss function that comprehensively considers point prediction accuracy and interval prediction quality. By introducing a NASA Score-aware regularization term, it optimizes the calibration and practicality of the prediction intervals while maintaining prediction accuracy.
3. We achieved adaptive adjustment of prediction intervals: We proposed an interval adjustment mechanism based on learnable scaling parameters, capable of dynamically adjusting the width of prediction intervals according to RUL values, better reflecting uncertainty characteristics at different life stages.
4. We established a complete evaluation system: We constructed a comprehensive evaluation framework including multiple dimensions such as point prediction accuracy, interval coverage, and interval width, providing a comprehensive and objective standard for evaluating the performance of probabilistic prediction models.

The structure of the rest of this paper is arranged as follows: Section 2 outlines the research status in the field of RUL and RUL uncertainty estimation. Section 3 introduces the proposed method. Section 4 analyzes the experimental dataset and conducts comparative experiments using relevant algorithms. Section 5 presents the conclusion of this paper and prospects for future work.

## 2. Related Work

Probabilistic prediction methods are mainly divided into two categories: parametric methods and non-parametric methods. Parametric methods assume that the data follows a specific probability distribution and achieve probabilistic prediction by estimating the distribution parameters; non-parametric methods do not rely on specific distribution assumptions and make predictions directly based on data characteristics [6]. For a long time, the main focus in the field of time-series forecasting has remained on point prediction, i.e., directly estimating specific point data, neglecting the application of probabilistic prediction and uncertainty quantification in time-series forecasting. In fact, researching uncertainty quantification technology is of great significance in the field of RUL prediction and is more suitable for decision-makers in certain application scenarios. In recent years, many scholars have conducted research in this area. For example, Wang et al. [2] proposed a distribution-agnostic probabilistic few-shot learning method, achieving probabilistic modeling of failure modes and RUL through multi-modal Bayesian Model–Agnostic Meta-Learning (MBMAML). Li et al. [7] proposed the SeqBAST model, which uses a Bayesian attention mechanism and state transition modules to quantify the uncertainty of RUL prediction. Ding et al. [8] proposed an RUL prediction method based on a Gaussian Process Autoregressive-Variational Autoencoder (GPVAE) framework. This method can quantify prediction uncertainty and decompose it into epistemic uncertainty and aleatoric uncertainty, further quantifying the epistemic uncertainty of RUL-related features. Liu et al. [9] proposed a semi-supervised learning method for quantifying uncertainty and providing interval prediction for equipment RUL. By combining labeled and unlabeled data, it improves the reliability and accuracy of prediction results. Wang et al. [10] proposed an RUL prediction method based on a Bayesian Large-Kernel Attention Network. This method effectively captures long-term dependencies and spatial features in bearing condition monitoring data by introducing a large-kernel attention mechanism, while simultaneously using the Bayesian framework to comprehensively quantify prediction uncertainty. Kordestani et al. [11] proposed a new condition-based monitoring and fusion approach for bearing life prediction, ensuring that the prediction results have bounded uncertainty.

In addition to the traditional probability methods like Bayesian and Gaussian mentioned above, in recent years, with the continuous development of deep learning technology, recurrent neural networks and their variants such as Long Short-Term Memory and Gated Recurrent Units have been widely used in time-series forecasting tasks due to their excellent sequence modeling capabilities. These models can effectively capture long-term dependencies in time series, laying the foundation for accurate prediction, and have played a significant role in uncertainty prediction for time series [12]. Among them, LSTM effectively solves the gradient vanishing and explosion problems during RNN training by introducing gating mechanisms, enabling it to learn long-term dependencies [13]. For example, Chen et al. [14] proposed an RUL prediction interval estimation method based on bidirectional LSTM, quantifying prediction uncertainty by combining improved fuzzy C-means clustering and statistical analysis. Wei et al. [15] proposed a lithium-ion battery RUL prediction method combining Gated Recurrent Unit and Monte Carlo Dropout. By extracting the charging voltage saturation time as a health indicator and using Monte Carlo sampling to quantify prediction uncertainty, it can generate a reliable 95% confidence interval while providing accurate point prediction. Catelani et al. [16] and Yang et al. [17] proposed a hybrid method based on a recurrent neural network and state space estimation to improve the accuracy and precision of lithium-ion battery RUL prediction.

Some specially designed neural network structures in deep learning, such as Convolutional Neural Networks [18], attention mechanisms [19,20], Graph Convolutional Networks [21], and Transformer architectures [22], have also been applied in RUL uncertainty prediction. For example, Zhu et al. [23] proposed a Recurrent Graph Convolutional Network with uncertainty estimation for RUL prediction. This network combines graph learning modules, correlation mining modules, and fusion modules to capture spatiotemporal correlations in condition monitoring data and simultaneously estimate prediction uncertainty. Jiang et al. [24] built an RUL prediction model combining a Convolutional Neural Network and a Long Short-Term Memory network to provide confidence intervals for rolling bearing RUL prediction. Cao et al. [25] proposed a parallel Gated Recurrent Unit model with a dual-stage attention mechanism combined with uncertainty quantification. By introducing a dual-stage attention mechanism, the model can more effectively capture key features in time-series data while quantifying uncertainty in the prediction process. Liu et al. [26] also proposed an RUL prediction method with a dual-stage attention mechanism and achieved good prediction results.

The fusion of probability models such as Bayesian models and Gaussian models with deep learning also provides a powerful theoretical framework and tools for RUL uncertainty prediction [27]. The core advantage of such models is that they retain the powerful nonlinear feature extraction and sequence modeling capabilities of deep neural networks, enabling automatic learning of health indicators and aging patterns from complex time series. At the same time, they introduce the probabilistic interpretation of Bayesian methods by treating the weights in the network as probability distributions rather than fixed values, thus naturally supporting the quantification of uncertainty in prediction results [28]. For example, Benker et al. [29] proposed using Bayesian deep learning models to estimate RUL. The results show that Bayesian deep learning models perform excellently in RUL estimation. Xu et al. [3] proposed an RUL prediction model combining the advantages of Deep Learning and Non-stationary Gaussian Process Regression to address high-dimensional input features, sensor data noise, and time dependence between system degradation and RUL prediction. Lin et al. [30] and Caceres et al. [31] proposed a Bayesian deep learning-based RUL prediction framework integrating uncertainty quantification and calibration. The framework uses concrete dropout technology to automatically optimize dropout probability to capture epistemic uncertainty and applies a Gaussian distribution to model outputs to quantify aleatoric uncertainty. Nguyen et al. [32] proposed a probabilistic deep learning-based method for uncertainty quantification of RUL in multi-component systems. By combining deep learning models with a probabilistic framework, this method can simultaneously predict the RUL of multi-component systems and quantify their uncertainty.

Quantile regression is a typical non-parametric method. It directly estimates conditional quantiles by optimizing the quantile loss function, avoiding biases that may arise from distribution assumptions. Therefore, quantile regression provides a natural technical framework for probabilistic interval prediction [33]. It can simultaneously estimate multiple quantiles, thereby constructing prediction intervals for RUL. The existing literature describes various methods for implementing quantile regression [34,35]. Traditional quantile regression methods are mostly based on linear models and have difficulty handling complex nonlinear relationships. Deep quantile regression combines deep learning with quantile regression, utilizing the powerful representation ability of neural networks to learn complex nonlinear quantile relationships. Such methods can simultaneously output predicted values at multiple quantile points by minimizing the quantile loss function, thereby achieving probabilistic interval prediction [36]. For example, Frizzo et al. [37] proposed a method combining State Space Models and Simultaneous Quantile Regression for probabilistic RUL prediction, which can improve computational efficiency and provide reliable uncertainty quantification. ZHANG et al. [38] proposed a distributed remaining useful life prediction method based on quantile regression and deep learning, combined with quantile Huber loss for optimization, which can directly output the cumulative distribution function of RUL to quantify prediction uncertainty. Wu et al. [39] proposed a lithium-ion battery life prediction model based on a DAE-CNN-BiGRU fusion architecture and quantile regression, using quantile regression to quantify prediction uncertainty and generate reliable confidence intervals. Chen et al. [40] achieved accurate prediction and uncertainty quantification of equipment failure time by combining deep learning quantile regression with kernel density estimation. Mao et al. [41] proposed an RUL prediction method integrating time-series feature extraction and Conformalized Quantile Regression. CQR introduces conformal prediction theory on the basis of quantile regression to generate prediction intervals with statistical guarantees. Tian et al. [42] proposed an RUL prediction method combining reliable degradation indicators and Temporal Convolution Network quantile regression. It uses the causal convolution and dilated convolution structure of a TCN to efficiently capture long- and short-term dependencies in time-series data and finally generates RUL prediction intervals with probabilistic significance through quantile regression. Cui et al. [43] proposed an ensemble deep learning framework that improves the reliability of prediction intervals and the accuracy of probability density estimation through the combination of non-crossing quantile regression and kernel density estimation. Bhatnagar et al. [44] combined deep learning with quantile regression for model calibration, using deep learning models to capture complex nonlinear features and using quantile regression to quantify prediction uncertainty, thereby improving the reliability and practicality of the model in time-series prediction. Tambuwal et al. [45] combined unsupervised learning to identify outliers by modeling the distribution of time-series data at different quantiles. The core is to use quantile regression to capture the fluctuation range of the data, detecting extreme points that deviate from the normal distribution without requiring labels. Hu et al. [46] achieved multi-quantile joint prediction and uncertainty quantification for energy time series by integrating the nonlinear modeling capability of neural networks with the quantile estimation characteristics of quantile regression, combined with monotonicity constraint optimization technology.

The model proposed in this paper integrates the temporal modeling capability of LSTM, the feature selection capability of the attention mechanism, and the uncertainty quantification capability of quantile regression. Compared with the methods in the aforementioned literature, it has the following innovations: First, compared with traditional quantile regression methods, the method in this paper automatically learns feature representations through deep learning networks, does not require manual feature design, and can better capture complex patterns in sensor data. Second, compared with basic deep quantile regression methods, this paper introduces an attention mechanism and a NASA Score optimization module, designing learnable scaling parameters to adaptively adjust prediction intervals, avoiding high penalties for early predictions, and thereby optimizing the accuracy and interpretability of prediction intervals.

## 3. Method

### 3.1. Problem Definition

For a given multivariate sensor sequence of length T, $X_{1:T}=\{x_{1},x_{2},\ldots ,x_{T}\}$, where the feature vector $x_{t}\in R^{d}$ at each time step contains d-dimensional sensor time-series signals, RUL is defined as:

$$y=T_{failure}-T_{current}\geq 0$$

Traditional point prediction methods mainly learn the mapping $f:X_{1:T}\mapsto \hat{y}$ and cannot quantify prediction uncertainty. The quantile regression framework adopted in this paper does not require prior assumptions about the error distribution. For any quantile level *τ*, the conditional quantile function can be defined as:

$$Q_{\tau }\;(y|X_{1:T}\;)=\inf\{y:F(y|X_{1:T}\;)\geq \tau \}$$

where $F(y|X_{1:T}\;)$ is the conditional cumulative distribution function. Quantile regression estimates the conditional quantile by minimizing an asymmetrically weighted loss function:

$$Q_{\tau }\;(y|X_{1:T}\;)=\arg\underset{\theta }{\min}\;E[\rho_{\tau }\;(y-f_{\theta }\;(X_{1:T}))]$$

where the quantile loss function $\rho_{\tau }\left(.\right)$ is defined as:

$$\rho_{\tau }\left(u\right)=\left\{\begin{array}{l} \tau u,\;\;\;\;\;\;\;\;\;\;\;\;\mathrm{if}\;u\geq 0\\ \left(\tau -1\right)u\;\;\;\;\mathrm{if}\;u<0 \end{array}\right.\;\;=u\left(\tau -I_{u}<0\right)$$

when the predicted value is lower than the true value (u > 0), the weight is τ; when the predicted value is higher than the true value (u < 0), the weight is τ−1.

This paper selects three quantile levels with clear physical meaning:

τ∈T={0.1,0.5,0.9}

corresponding to the pessimistic case, median estimate, and optimistic case of RUL prediction, respectively. Based on this, a deep quantile regression model is constructed:

$$f_{\theta }:X_{1:T}\mapsto \left[{\hat{y}}_{0.1},\;{\hat{y}}_{0.5},\;{\hat{y}}_{0.9}\right]$$

where θ represents the model parameters. These three quantile predictions constitute a complete probabilistic prediction, where ${\hat{y}}_{0.5}$ is the point estimate, and the interval $\left[{\hat{y}}_{0.1},\;\;{\hat{y}}_{0.9}\right]$ constitutes the 80% confidence interval for the RUL prediction, i.e.:

$$P\left({\hat{y}}_{0.1}\leq {\hat{y}}_{0.9}\left|X_{1:T}\right.\right)\approx 0.8$$

The overall optimization objective is to minimize the loss functions for the three quantile levels simultaneously:

$$L(\theta )=\frac{1}{N}\sum_{i=1}^{N}\sum_{\tau \in T}\rho_{\tau }\left(y_{i}-{\hat{y}}_{i,\tau }\right)+\lambda \Omega (\theta )$$

where Ω(θ) is the regularization term and λ is the regularization coefficient.

Table 1 is the nomenclature table, which systematically defines all mathematical symbols, variables, and indexes used throughout the formulations, including the quantile levels, loss functions, scaling parameters, and model hyperparameters.

### 3.2. Model Architecture

#### 3.2.1. Overall Architecture

**Figure 1:**
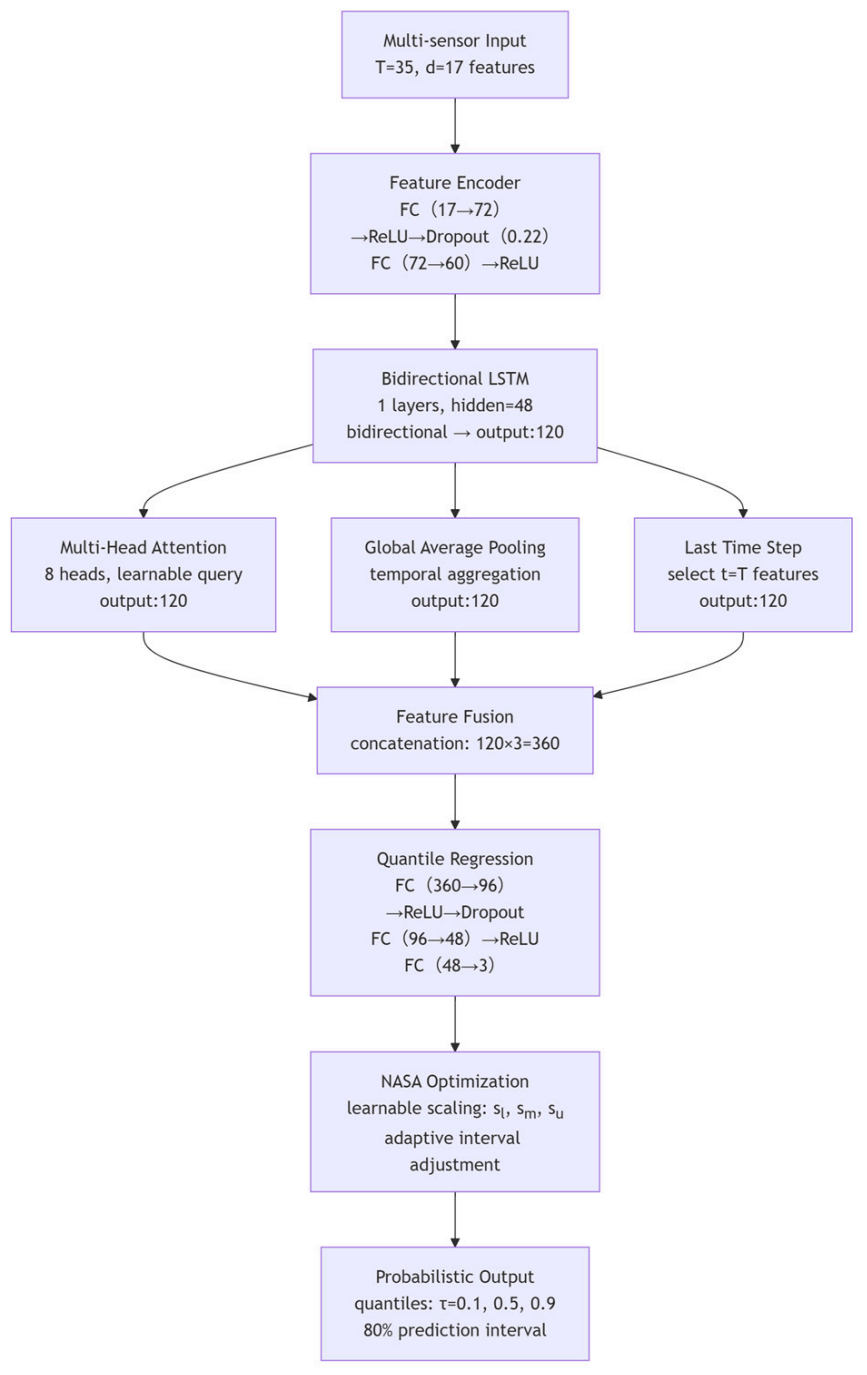
Overall architecture of quantile attention-based model.

The proposed quantile attention-based model adopts a deep learning-based hierarchical hybrid architecture. The overall framework is shown in Figure 1. The overall architecture follows the design concept of hierarchical encoding and feature fusion, designed according to the data flow of “Input Encoding → Temporal Modeling → Multi-scale Feature Extraction → Feature Fusion → Probabilistic Output”. Through multi-level feature learning and uncertainty quantification, it achieves RUL point prediction while simultaneously extending to probabilistic interval prediction.

The model takes multidimensional sensor time-series data as input. First, feature encoding is performed through a two-layer fully connected network. Then, BiLSTM is used to capture temporal dependencies, and a multi-head attention mechanism is introduced to focus on key degradation stages. A feature fusion layer integrates the attention output, global average features, and the last time step features to form a 360-dimensional comprehensive temporal representation of the system. Then, a quantile regression network outputs the three quantile predictions (10%, 50%, 90%). On this basis, a NASA optimization module is designed to adaptively adjust the prediction interval through learnable scaling parameters, effectively reducing the penalty loss for early predictions. The overall system input and output are as follows: let the input multivariate time-series data be $X=\{x_{1}\;,x_{2}\;,\ldots ,x_{T}\;\}\in R^{T\times d}$, where T is the time step length and d is the feature dimension. The model’s goal is to learn a mapping function, outputting the three conditional quantile predictions:

$$\hat{y}=[{\hat{y}}_{0.1},{\hat{y}}_{0.5},{\hat{y}}_{0.9}]=f(X)$$

forming the 80% confidence interval $\left[{\hat{y}}_{0.1},{\hat{y}}_{0.9}\right]$, where ${\hat{y}}_{0.5}$ is the point estimate.

#### 3.2.2. Feature Encoding Layer and Temporal Modeling Layer Design

In the system model architecture, the feature encoding layer is responsible for transforming raw data into high-dimensional features, using a two-layer fully connected network structure:

$$Z^{\left(1\right)}=W_{1\;}X+b_{1}\;$$

$$H^{\left(1\right)}=\max(0,\;\;Z^{\left(1\right)})\;\left(\mathrm{ReLU}\;\mathrm{activation}\;\mathrm{function}\right)$$

$$Z^{\left(2\right)}=W_{2\;}H^{\left(1\right)}+b_{2}\;$$

$$H=\max(0,\;\;Z^{\left(2\right)})\;$$

where $W_{1}\in R^{72\times 17}$, $b_{1}\in R^{72}$, $W_{2}\in R^{60\times 72}$, and $b_{2}\in R^{60}$. The first layer expands the feature dimension from 17 to 72, and the second layer compresses it to 48 dimensions. This “expansion-compression” strategy helps capture complex interactions between time-series signal features. Dropout regularization is used to randomly discard neurons with a certain probability:

$$H_{dropout\;}=H⊙M$$

where $M\in {\left\{0,\;1\right\}}^{60}\;$ is a Bernoulli random vector, effectively preventing overfitting.

The temporal modeling layer consists of a bidirectional LSTM network, including forward and backward LSTMs that process the time series in forward and reverse order, respectively. The forward LSTM and backward LSTM have the same structure, but the backward LSTM processes the sequence in reverse chronological order. Therefore, the final bidirectional output of the BiLSTM is:

$$h_{t}^{\mathrm{bi}}=[\vec{h_{t}};\;\overset{\leftarrow }{h_{t}}\;]\in R^{120}$$

Stacking two LSTM layers further enhances the model’s representation ability:

$$h_{t}^{\left(2\right)}={\mathrm{LSTM}}^{\left(2\right)}(h_{t}^{\left(1\right)})$$

#### 3.2.3. Attention Mechanism Design

**Figure 2:**
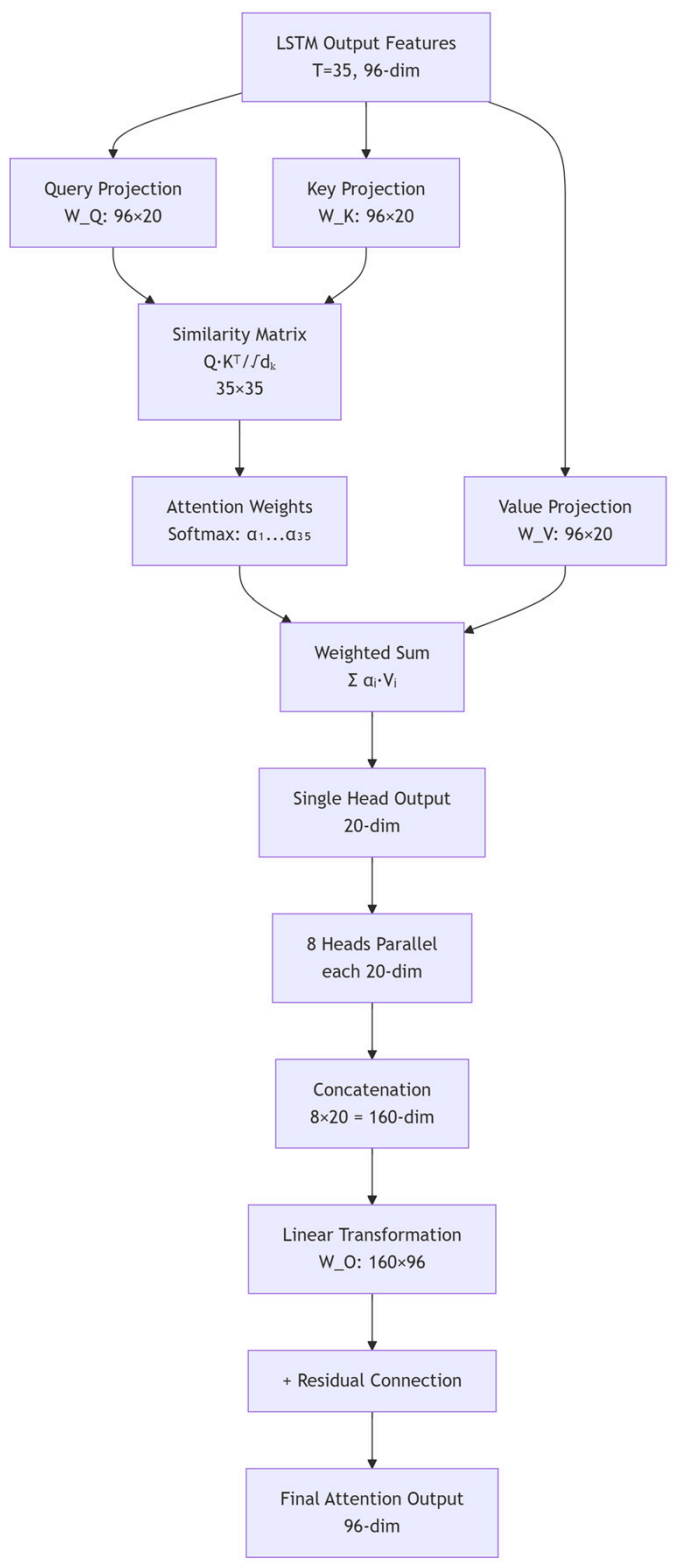
Architecture of the designed attention mechanism.

To overcome the limitation of traditional LSTM models treating all time steps equally and to better capture long-term dependencies in time series, we designed a multi-head attention mechanism that maps the input to multiple subspaces for parallel computation. Its architecture is shown in Figure 2.

Different from traditional self-attention, we introduced a learnable query vector $q\in R^{d_{k}}\;$, optimized automatically through the training process:

$$Q=q\cdot 1_{1\times T\;}\in R^{T\times d_{k}}\;$$

This design is optimized for the characteristics of the RUL prediction task and reduces the number of parameters and computational complexity. The multi-head attention mechanism maps the input to multiple subspaces for parallel computation. For the *i*-th attention head:

$$Q_{i}\;\;{=\mathrm{HW}}_{i}^{Q}\;,\;\;K_{i}\;{=\mathrm{HW}}_{i}^{K}\;,\;\;V_{i\;}{=\mathrm{HW}}_{i}^{V}\;$$

$${\mathrm{head}}_{i}\;=\mathrm{softmax}\left(\frac{Q_{i}K_{i}^{T}}{\sqrt{d_{k}}}\right)V_{i}\;$$

where $W_{i}^{Q}$ and $W_{i}^{K}\in R^{d_{\mathrm{mod}el}\times d_{k}}\;$, $W_{i}^{V}\in R^{d_{\mathrm{mod}el}\times d_{v}}\;$, and $d_{k}\;=\;d_{v}\;=\;d_{model}/h\;=\;20\;$.

To capture different types of dependencies, a multi-head attention mechanism is used:

$${\mathrm{head}}_{i}\;=\mathrm{Attention}({\mathrm{QW}}_{i}^{Q}\;,\;\;{\mathrm{KW}}_{i}^{K}\;,\;\;{\mathrm{VW}}_{i}^{V}\;)$$

the multi-head mechanism allows the model to attend to information from different representation subspaces at different positions, potentially capturing diverse aspects of the sensor signals. All head outputs are then concatenated and linearly transformed to produce the final attended representation. The final multi-head output is fused through concatenation and linear transformation:

$$\mathrm{MultiHead}=\mathrm{Concat}({\mathrm{head}}_{1}\;\;,\;\ldots \;,\;head_{8}\;)W^{O}$$

where $W^{O}\in R^{8d_{v}\times d_{\mathrm{model}}}$ is the output projection matrix.

The combination of the attention mechanism and bidirectional LSTM forms a complementary advantage: LSTM is good at capturing local temporal patterns, while the attention mechanism provides global context information, jointly building a multi-level temporal understanding capability. This design enables the model to more accurately identify turning points in degradation trends, improving the timeliness and reliability of predictions.

Regarding potential computational concerns, we note that for the FD001 dataset, the sequence length is set to 35 time steps (as listed in Table 2), making the quadratic complexity of self-attention ($O\left(L^{2}\right)\;with\;L\;=\;35$) practically negligible. Moreover, RUL prediction typically involves offline training, where prediction accuracy is the primary concern rather than training parallelism.

#### 3.2.4. Multi-Objective Optimization Strategy

Aiming at the multiple objective requirements of RUL probabilistic interval prediction, we designed a comprehensive optimization strategy to balance three key dimensions: point prediction accuracy, interval coverage quality, and engineering practicality. First, the quantile regression loss function serves as the core of the optimization framework. By applying asymmetric penalties to prediction errors in different directions, it achieves accurate estimation of conditional quantiles and is the basic optimization objective of the model. For the three target quantiles $T\;\in \;\left\{0.1,\;0.5,\;0.9\right\}$, the overall quantile loss of the model is:

$$L_{quantile}\;=\;\frac{1}{N}\sum_{i=1}^{N}\sum_{\tau \in T}\rho_{T}\left(y_{i}\;-\;{\hat{y}}_{i,T}\right)$$

where $T\;=\;\left\{0.1,\;0.5,\;0.9\right\}$, and N is the number of samples.

Aiming at the specific evaluation standard of the CMAPSS dataset, this paper introduces a penalty mechanism guided by the NASA Score function. The original NASA Score function is:

$$\mathrm{Score}(d)=\left\{\begin{array}{l} \exp(d/10)-1\;,\;\;\;\;\;\;\mathrm{if}\;d\geq 0\\ \exp(-d/13)-1\;,\;\;\;\mathrm{if}\;d<0 \end{array}\right.$$

here, $d=\hat{y}-y$. This function imposes an exponential penalty for underestimating RUL. For this purpose, an early prediction penalty term is designed:

$$L_{quantile}\;=\;\frac{1}{N}\sum_{i=1}^{N}\max\left(0,\;y_{i}-{\hat{y}}_{i,\;0.5}\right)\;\cdot \;w\left(y_{i}\right)$$

The weight function $w\left(y_{i}\right)$ dynamically adjusts the penalty intensity according to the true RUL value:

$$w\left(y_{i}\right)=\;\frac{1}{1+\exp(-k\;(y_{i}-y_{\mathrm{threshold}}\;))}$$

where $y_{\mathrm{threshold}}\;=\;50$ is the penalty threshold and k = 0.1 controls the transition smoothness. This design ensures stronger penalties are applied at the end of life.

On the other hand, to improve the statistical properties of the prediction intervals, a joint regularization for coverage and interval width is introduced:

$$L_{\mathrm{interval}}\;=\;\lambda_{c}\;\cdot \;|\mathrm{Coverage}-0.8|\;+\lambda_{w}\;\cdot {\mathrm{Width}}_{\mathrm{norm}}\;$$

where the coverage is calculated as:

$$\mathrm{Coverage}=\frac{1}{N}\sum_{i=1}^{N}I\left\{y_{i}\in \left[{\hat{y}}_{i,0.1},{\hat{y}}_{i,0.9}\right]\right\}$$

The normalized interval width is:

$${\mathrm{Width}}_{\mathrm{norm}}=\frac{1}{N}\sum_{i=1}^{N}\frac{{\hat{y}}_{i,0.9}-{\hat{y}}_{i,0.1}}{y_{i}+\varepsilon }$$

where ε = 1 prevents division by zero errors.

Combining the above, the overall optimization objective of the model is the weighted sum of the above loss functions and regularization terms:

$$L_{\mathrm{total}}\;=\;L_{\mathrm{quantile}}\;+\;\lambda_{1}L_{\mathrm{early}}\;+\;\lambda_{2}L_{\mathrm{interval}}\;+\;\lambda_{3}{\left\|\Theta \right\|}_{2}^{2}\;$$

where $\lambda_{1}$ balances point prediction and early prediction penalty, $\lambda_{2}$ controls the strength of interval quality regularization, and $\lambda_{3}$ is the weight decay coefficient.

#### 3.2.5. Adaptive Interval Adjustment Mechanism

Aiming at the rigid interval limitation problem of traditional quantile regression in engineering applications, we designed an adaptive interval adjustment mechanism based on learnable parameters. This mechanism dynamically scales the prediction interval, effectively balancing statistical characteristics and engineering requirements as follows.

First, obtain the base predicted values through the deep quantile regression network:

$${\hat{y}}_{\mathrm{base}}=f_{\theta }\left(h_{\mathrm{fused}}\right)=[{\hat{y}}_{0.1}^{\mathrm{base}},\;{\hat{y}}_{0.5}^{\mathrm{base}},\;{\hat{y}}_{0.9}^{\mathrm{base}}]$$

where the base prediction interval width is:

$$W_{\mathrm{base}}={\hat{y}}_{0.9}^{\mathrm{base}}\;-\;{\hat{y}}_{0.1}^{\mathrm{base}}$$

Traditional methods directly use this interval, which can easily lead to insufficient adaptability to practical application scenarios. We introduce a learnable scaling parameter vector $s=[s_{l},s_{m},s_{u}]\in R^{3}$ on this basis, corresponding to the adjustment factors for the lower quantile, median, and upper quantile, respectively. The parameter initialization strategy is:

$$s_{l}^{\left(0\right)}\;\;=\;1.0\;,\;\;s_{m}^{\left(0\right)}=1.0\;,\;\;s_{u}^{\left(0\right)}\;\;=1.2$$

This initialization favors conservative estimation, especially expanding the upper quantile to avoid early prediction penalties. The scaling operation is achieved by the following formulas:

$$\Delta_{l}={\hat{y}}_{0.5}^{\mathrm{base}}-{\hat{y}}_{0.1}^{\mathrm{base}}$$

$$\Delta_{u}={\hat{y}}_{0.9}^{\mathrm{base}}-{\hat{y}}_{0.5}^{\mathrm{base}}$$

$${\hat{y}}_{0.1}^{\mathrm{base}}=\;{\hat{y}}_{0.5}^{\mathrm{base}}\;-\;s_{l}\cdot \Delta_{l}$$

$${\hat{y}}_{0.5}\;=\;s_{m}\cdot {\hat{y}}_{0.5}^{\mathrm{base}}$$

$${\hat{y}}_{0.9}\;=\;{\hat{y}}_{0.5}^{\mathrm{base}}\;+\;s_{u}\cdot \Delta_{u}$$

The scaling parameters are not fixed but are dynamically adjusted according to the predicted RUL value:

$$s_{l}\;(y_{\mathrm{pred}}\;)=s_{l}^{(0)\;}+\alpha_{l}\;\cdot \sigma (\beta_{l}\;\cdot (y_{\mathrm{pred}}\;-y_{\mathrm{threshold}}\;))$$

$$s_{u}\;(y_{\mathrm{pred}}\;)=s_{u}^{(0)\;}+\alpha_{u}\;\cdot \sigma (\beta_{u}\;\cdot (y_{\mathrm{pred}}\;-y_{\mathrm{threshold}}\;))$$

where $\sigma (x)=1/(1+e^{-x})$ is the sigmoid function and $y_{\mathrm{threshold}}$ is the adjustment threshold. When the predicted RUL is small, $s_{u}$ is automatically increased to provide a more conservative interval estimate.

To prevent the scaling parameters from deviating excessively and causing a decrease in interval quality, a constraint term based on statistical characteristics is introduced:

$$L_{\mathrm{constraint}}\;=\lambda_{\mathrm{width}}\;\cdot \max{\left(0\;,\;W_{\mathrm{norm}}\;-W_{\max}\;\right)}^{2}+\lambda_{\mathrm{ratio}}\;\cdot {\left(\frac{\Delta_{u}}{\Delta_{l}}\;-\;r_{\mathrm{target}}\;\right)}^{2}$$

where $W_{\mathrm{norm}}\;=\;\left({\hat{y}}_{0.9}\;-\;{\hat{y}}_{0.1}\right)/{\hat{y}}_{0.5}$ is the normalized interval width, $W_{\max}$ is the maximum allowed width, and $r_{\mathrm{target}}$ is the target upper/lower interval ratio.

During training, Monte Carlo simulation is used to evaluate the NASA Score performance of different scaling strategies:

$$E[\mathrm{Score}]=\frac{1}{M}\sum_{m=1}^{M}\mathrm{NASA}({\hat{y}}^{\left(m\right)}-y_{\mathrm{true}})$$

where ${\hat{y}}^{\left(m\right)}∼N({\hat{y}}_{0.5},({\hat{y}}_{0.9}-{\hat{y}}_{0.1})/3.92)$ generates samples based on the normal distribution assumption. The optimization objective combines the score expectation and interval quality:

$$L_{\mathrm{scale}}\;=E[\mathrm{Score}]+\gamma \cdot L_{\mathrm{constraint}}\;$$

## 4. Experiments

### 4.1. Experimental Data

**Figure 3:**
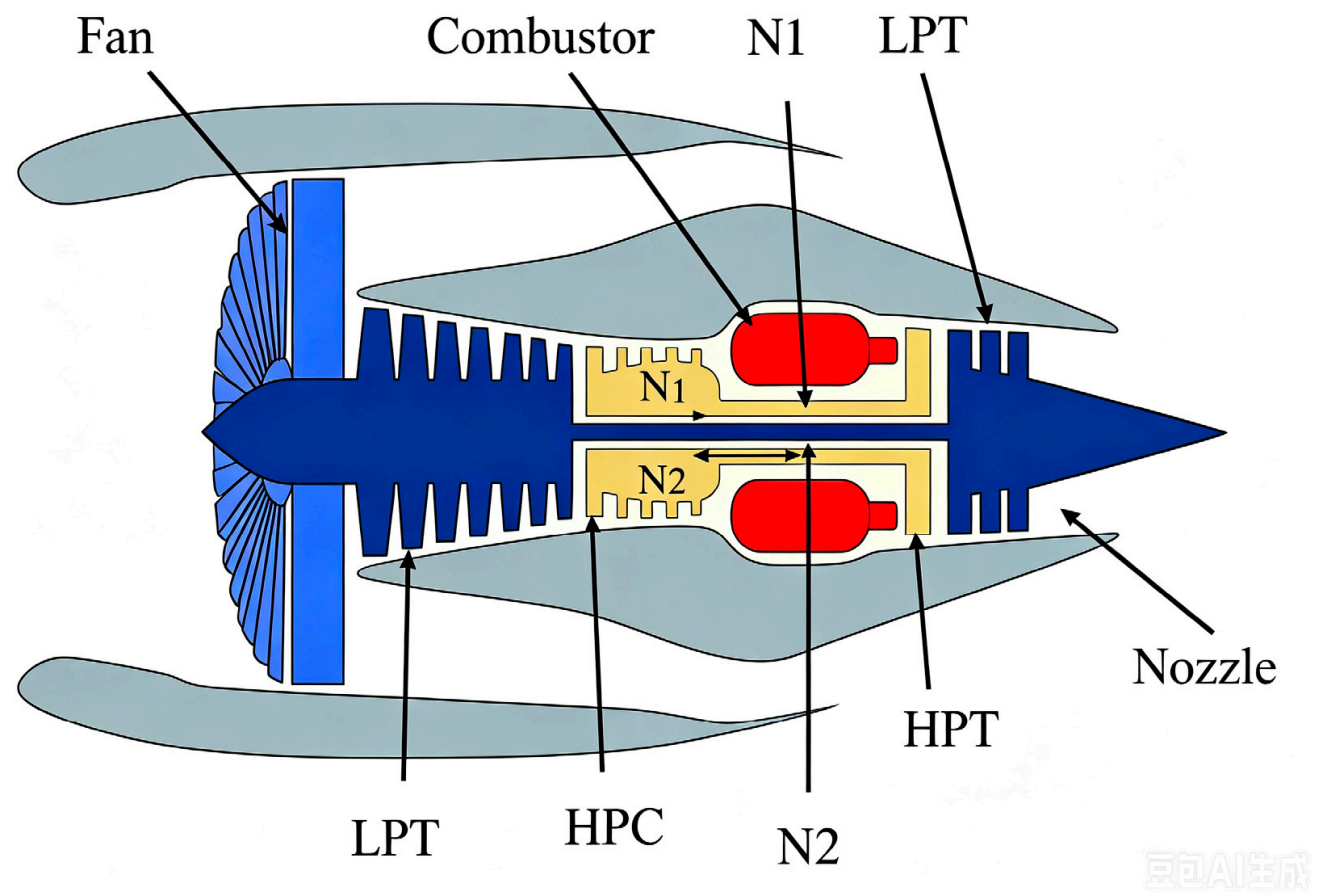
C-MAPSS aero-engine structure.

We used the NASA open-source C-MAPSS-based aero-engine simulation time-series dataset [47] to verify the effectiveness of the proposed model and method. Figure 3 shows a schematic diagram of the aero-engine structure based on C-MAPSS, including components such as the fan, combustion chamber, high/low-pressure compressor, turbine, and nozzle.

The C-MAPSS dataset contains four sub-datasets, each corresponding to different operating conditions and failure modes. This paper uses the FD001 dataset as an example to conduct model effectiveness experiments and comparative experiments. The FD001 dataset contains: a training set: complete run-to-failure data for 100 engines; a test set: partial run-to-failure data for 100 engines; data dimension: each time step contains 21 sensor measurements and 3 operational setting parameters; operating cycles: each engine runs until failure, with cycles between 128 and 362. Table 2 shows the statistical characteristics of the FD001 dataset.

### 4.2. Experimental Setup

#### 4.2.1. Evaluation Metrics

To comprehensively evaluate the model’s overall performance, this paper establishes a multi-dimensional evaluation index system, covering three aspects: point prediction accuracy, interval prediction quality, and engineering application value. Among them, point prediction accuracy metrics mainly evaluate the accuracy of the median prediction:

Root Mean Square Error (RMSE):

$$\mathrm{RMSE}=\sqrt{\frac{1}{N}\sum_{i=1}^{N}{\left(y_{i}-{\hat{y}}_{i,0.5}\right)}^{2}}$$

Interval prediction quality metrics evaluate the statistical properties of the probabilistic prediction, including interval coverage and average interval width:

$$\mathrm{Coverage}=\frac{1}{N}\sum_{i=1}^{N}Iy_{i}\in [{\hat{y}}_{i,0.1},{\hat{y}}_{i,0.9}]$$

$$\mathrm{MIW}=\frac{1}{N}\sum_{i=1}^{N}\left({\hat{y}}_{i,0.9}-{\hat{y}}_{i,0.1}\right)$$

Engineering application metrics focus on the model’s practicality in real scenarios. Considering the different damage levels of early and late RUL predictions for the system, the NASA Score is used to evaluate the engineering application value of the model’s point prediction:

$$\mathrm{NASA}\;\mathrm{Score}=\sum_{i=1}^{N}\left\{\begin{array}{l} \exp(\frac{d_{i}}{10})-1,\;\;\;\;\;\;\;\;d_{i}\geq 0\;\;\\ \exp(-\frac{d_{i}}{13})-1,\;\;\;\;\;\;d_{i}<0 \end{array}\right.$$

where $d=\hat{y}-y$.

#### 4.2.2. Model Settings and Training

The model’s network structure uses a two-layer bidirectional LSTM architecture, along with a multi-head attention mechanism, where each attention head has a corresponding dimension. The feature encoder uses a two-layer fully connected network with dimensions of 72 and 48, with the ReLU activation function and dropout regularization to prevent overfitting. In terms of training strategy, aiming at the characteristics of the RUL prediction task, we adopted a phased training strategy. The initial stage uses a larger learning rate for coarse adjustment, and the learning rate is gradually reduced in the later stage for fine optimization. The AdamW optimizer is chosen, and its built-in weight decay mechanism helps improve model generalization ability. The sequence length is determined to be 30 time steps. This length is sufficient to capture the equipment’s degradation trend while avoiding the computational burden caused by overly long sequences. Some of the model’s hyperparameter configurations are shown in Table 3. In terms of experimental environment configuration, all experiments in this paper were completed on the Kaggle cloud computing platform [48]. The hardware configuration uses the Kaggle Notebooks standard environment, equipped with a 2-core CPU, 13 GB of memory, and an NVIDIA T4 GPU. The software environment is based on its pre-configured Python 3.7 and PyTorch 1.12 framework.

The hyperparameters listed in Table 3 were determined through Bayesian optimization using the Tree-structured Parzen Estimator (TPE) algorithm implemented in the Optuna library. The resulting configuration (d_model = 48, nhead = 8, num_layers = 1, dropout = 0.22) consistently yielded superior performance across multiple validation splits. In particular, we found that: (i) a single LSTM layer achieves better generalization than two layers, as deeper LSTM tends to overfit on the limited training data; (ii) eight attention heads provide a favorable balance between representational capacity and computational cost, as each head captures distinct degradation patterns (e.g., long-term trends and short-term anomalies); (iii) the hidden dimension of 48 offers sufficient capacity for feature extraction while maintaining a lightweight model (112K parameters); and (iv) a moderate dropout rate of 0.22 effectively prevents overfitting without underfitting. This systematic approach ensures that our model achieves its optimal performance while maintaining computational efficiency for practical deployment.

### 4.3. Result Analysis

#### 4.3.1. Model Performance Effectiveness Verification

**Figure 4:**
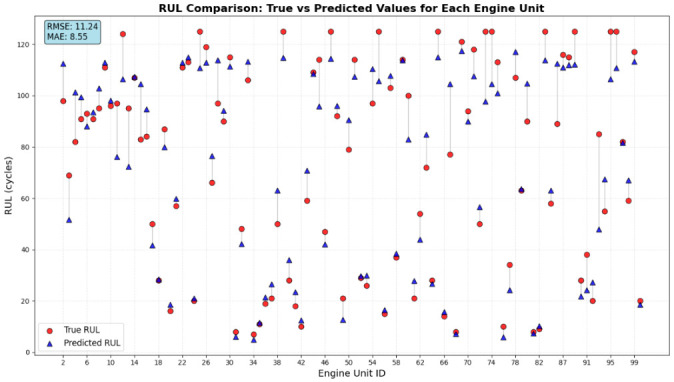
RUL comparison: true vs. predicted values for each engine unit.

To verify the model’s effectiveness, we conducted experiments on the C-MAPSS FD001 dataset. First, we tested the model’s point prediction accuracy. Figure 4 shows the model’s performance in RUL point prediction on the dataset. The model achieved results of RMSE = 11.24 and MAE = 8.55. Figure 4 intuitively shows the model’s RUL prediction effect for the 100 test engine units. It can be seen from the figure that the overall trend of the predicted values and the true values is relatively consistent. The gray connecting lines show the specific prediction deviation for each unit. Short lines indicate accurate predictions, while long lines indicate large errors. It can be seen from the figure that the prediction errors for most units are small, indicating that the model has high accuracy for RUL point prediction.

**Figure 5:**
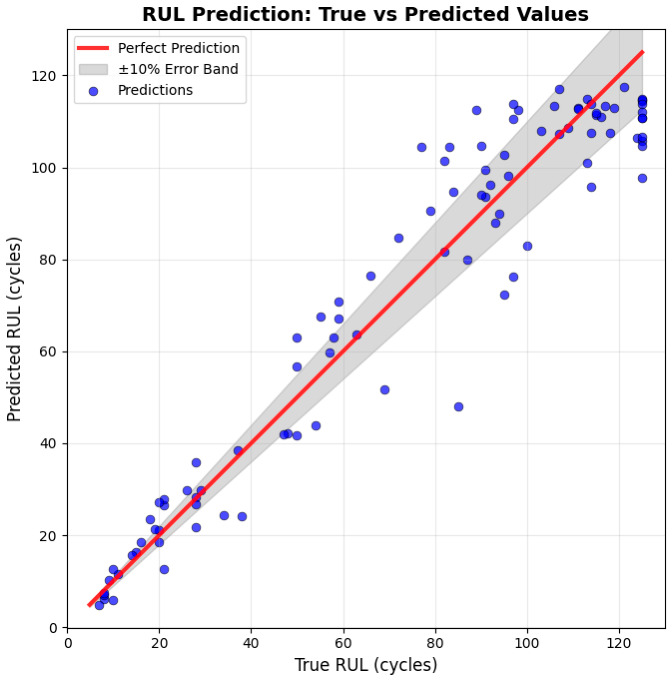
Scatter distribution of true vs. predicted values.

Figure 5 shows the scatter distribution of RUL predicted values versus true values, intuitively reflecting the model’s overall prediction accuracy. It can be seen from the figure that most data points are closely distributed near the red diagonal line, indicating that the model has good prediction accuracy. A considerable proportion of data points are contained within the gray shaded area, indicating that the model’s prediction effect within the acceptable error range is good. We can observe that in the low-RUL region, the prediction points are more concentrated and closer to the diagonal line, indicating that the model’s predictions are more accurate when approaching failure; while in the high-RUL region, the data points are relatively scattered, indicating a certain degree of prediction uncertainty. Some points fall outside the error band, mainly concentrated in the high-RUL region, which may reflect that the model’s learning of early degradation features is not sufficient.

**Figure 6:**
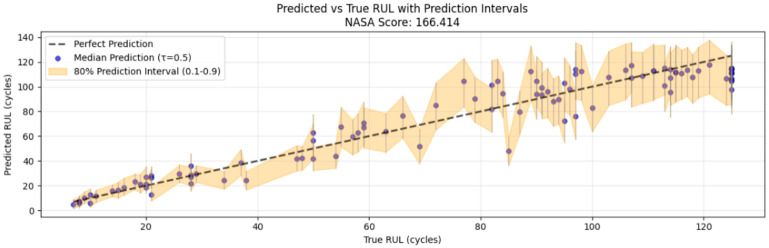
Predicted vs. true RUL with prediction intervals.

To verify the model’s ability for probabilistic interval prediction of RUL uncertainty, Figure 6 systematically demonstrates the model’s interval prediction performance by sorting according to the true RUL. It can be seen from the figure that the model’s RUL uncertainty prediction has several obvious characteristics: First, the prediction interval narrows significantly in the low-RUL region, indicating that the model is more certain about predictions near failure; while the interval is wider in the high-RUL region, reasonably reflecting the uncertainty of early degradation prediction. Second, the median prediction is basically distributed around the diagonal line throughout the entire RUL range, indicating that the model does not have significant systematic bias. However, slight prediction dispersion can be observed in the high-RUL region, with some points deviating far from the diagonal line, which may be due to relatively imperceptible early degradation features. It is worth noting that the estimated 80% prediction interval can effectively cover most of the true values, and the interval width shows reasonable adaptive adjustment with changes in RUL.

**Figure 7:**
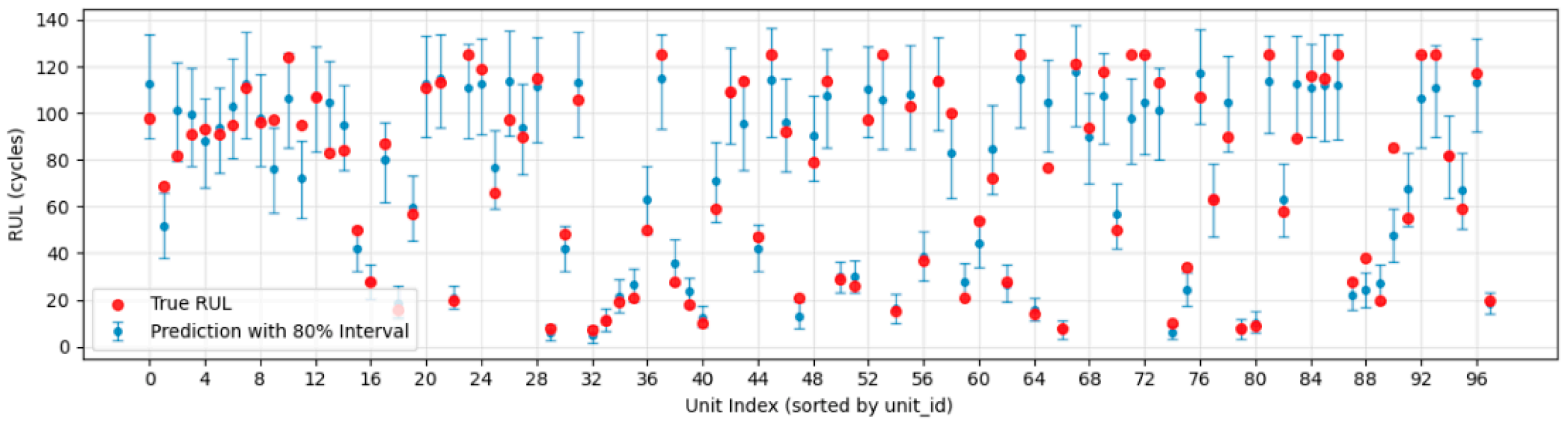
Coverage for predicted RUL intervals.

Figure 7 shows the model’s interval prediction effect sorted by engine unit ID. This figure clearly shows the correspondence between the 80% prediction interval for each test unit and the true RUL value. The model’s coverage is 0.867, and the interval width is 30.398. It can be seen from the figure that the prediction interval width remains relatively stable between different units, indicating that the model’s quantification of uncertainty is consistent. Most true values fall within the prediction interval, verifying the reliability of the interval estimation. The error distribution shows that the model does not have significant systematic bias, and the prediction interval can capture the fluctuation of the true RUL at different life stages. It is worth noting that for engine units with larger RUL, their prediction intervals are significantly wider than those for units with smaller RUL. This phenomenon is actually quite understandable because a smaller RUL indicates that the model has better captured the characteristics of its performance degradation process, so the model is more confident in its RUL prediction results, and thus its prediction interval is narrower.

**Figure 8:**
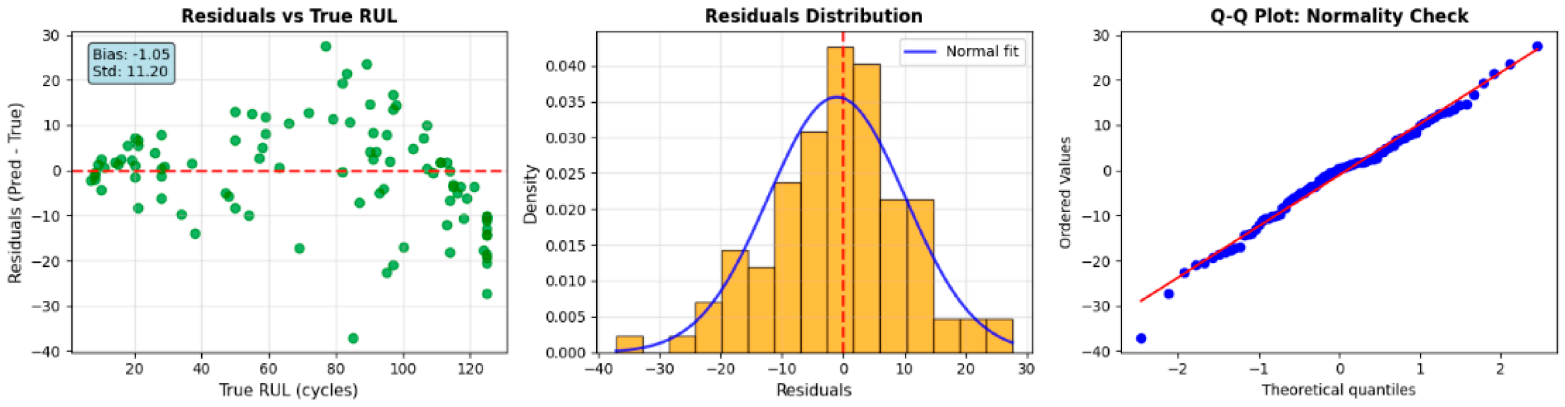
Residual analysis plot.

Figure 8 shows the error analysis of the model’s predictions, evaluating the model’s prediction performance from a statistical perspective. The residual scatter plot shows that the prediction errors are relatively evenly distributed across different RUL ranges, with Bias and Std being −1.05 and 11.2, respectively, and no obvious heteroscedasticity is observed, indicating that the model’s prediction stability is good at different life stages. However, a slight decrease in residual variance can be observed in the low-RUL region, which is expected—degradation trajectories become more obvious near failure, and prediction uncertainty naturally decreases. The residual distribution histogram shows an approximately normal distribution shape but is slightly right-skewed, indicating that the model has a slight tendency to overestimate. The Q-Q plot further verifies this finding: the residual points deviate from the theoretical normal line at both ends, with the right tail slightly longer, indicating the existence of a few large positive errors. This bias pattern may be related to the model’s over-sensitivity to certain degradation patterns. Error statistics show that the mean bias is close to zero, but the standard deviation is relatively significant, suggesting that although the model is unbiased overall, individual predictions have large fluctuations. The proportion of samples within the ±10% error range provides a practical indicator, reflecting the model’s reliability within the engineering acceptable accuracy range.

Table 4 presents the prediction results for different RUL intervals. The RMSE and MAE are not strictly increasing; they peak in the [60, 90] interval (18.377 and 15.365) and then decline in the final stage. Coverage rates for the four intervals are 0.92, 0.857, 0.615, and 0.943, with an overall coverage of 0.867, exceeding the target of 0.8. The prediction interval width expands from 11.758 to 40.918 as RUL increases, following the ‘narrow near, wide far’ principle, while the overall width of 30.398 maintains a reasonable balance between precision and coverage.

#### 4.3.2. Comparative Experiment Analysis

To comprehensively evaluate the performance of the proposed RUL uncertainty prediction model based on the attention mechanism and quantile regression on the FD001 dataset, this section compares it with various advanced RUL prediction methods. The comparison models cover traditional deep learning models, attention-mechanism-enhanced models, and the latest Transformer-based architectures. Evaluation metrics use the widely recognized RMSE and NASA Score in this field. The comparison results are shown in Table 5.

The following conclusions can be drawn from the table: In terms of prediction accuracy, the RMSE of the model in this paper is 11.245, which is lower than all the comparison models in the table, achieving the optimal performance. This indicates that the point prediction provided by this model is the most accurate, with the smallest deviation between predicted and true values. In terms of engineering practicality, the NASA Score of the model in this paper is 166.414, which is also better than all the comparison models, showing the best engineering application value. The advantage of this indicator shows that the model in this paper can effectively control the exponential high penalty caused by early prediction, which is crucial for actual predictive maintenance decisions.

In summary, the hybrid model integrating an LSTM–attention mechanism and quantile regression proposed in this paper achieves the best balance between prediction accuracy and engineering practicality. This not only verifies the effectiveness of the proposed architecture in temporal feature extraction and key information focusing but also proves the advantage of the quantile regression framework in generating uncertainty intervals with both accuracy and interpretability.

#### 4.3.3. Ablation Experiment Analysis

To verify the effectiveness of each component in the proposed LSTM–attention mechanism and quantile regression hybrid architecture, this section designs systematic ablation experiments. The experiments compare the performance of the full model with four ablation variants on the FD001 dataset. The results are shown in Table 6 and Table 7. From the overall performance perspective, the full model performs best in both core metrics, RMSE and NASA Score, while achieving a coverage close to the target under the premise of maintaining a reasonable interval width. In contrast, all ablation models show varying degrees of performance degradation, fully verifying the rationality of the full architecture design.

Key role of the attention mechanism: Removing the attention mechanism causes the NASA Score to increase by 79.6% and the RMSE to increase by 24.4%. This significant decline indicates that the attention mechanism plays an important role in focusing on key degradation features. It is worth noting that the interval coverage of this variant slightly improves, but this comes at the cost of a 19.8% increase in interval width, which is an overly conservative estimate and is not desirable in practical engineering applications.

Core value of LSTM temporal modeling: Removing the LSTM module has the most serious impact on performance, with the NASA Score worsening by 164.8% and the RMSE increasing by 30.8%. This result verifies the irreplaceability of LSTM in capturing long-term temporal dependencies in the equipment degradation process. Its gating mechanism can effectively learn the degradation patterns of time series.

Engineering significance of the NASA optimization module: Although removing the NASA optimization module has a relatively small impact on RMSE, the NASA Score significantly worsens by 93.5%. This difference highlights the value of this module in improving the model’s engineering practicality. The NASA optimization module effectively balances point prediction accuracy and engineering safety requirements by introducing learnable scaling parameters and an early prediction penalty mechanism, avoiding serious consequences that may be caused by early predictions.

Comprehensive advantage of the basic architecture: The basic quantile regression model performs the worst among all ablation variants, with the NASA Score worsening by 158.2% and the RMSE increasing by 44.9%. This model only retains the basic quantile regression framework, removing the three core components of LSTM temporal modeling, attention feature selection, and NASA optimization. Its poor performance conversely verifies the comprehensive advantage of the hybrid architecture proposed in this paper for the RUL uncertainty prediction task.

Interval prediction quality analysis: From the perspective of uncertainty quantification, the full model achieves the best balance between interval width and coverage. All ablation models show an increase in interval width. The Basic_QR model has the largest interval width, which is 48.5% larger than the full model. This indicates that although the simplified architecture may improve interval coverage, it comes at the cost of excessive prediction uncertainty, reducing the practical reference value of the prediction results.

#### 4.3.4. Efficiency Analysis

To evaluate the practical deployability of our model in industrial scenarios, we analyze its structural efficiency in terms of parameter scale, computational cost, inference time, and memory usage. All efficiency measurements were conducted on an NVIDIA T4 GPU (16 GB) with a batch size of 64.

As summarized in Table 6, our full model contains 112,326 trainable parameters, which is substantially smaller than typical deep learning models (e.g., ResNet-18 has ~11.7 million parameters). The number of floating-point operations (FLOPs) for a single forward inference is approximately 1.540 M, equivalent to 0.770 M Multiply-Accumulate operations (Mult-Adds).

The average inference time per engine unit is 1.66 ms on a single sample, which is negligible for offline prognostic tasks and sufficiently fast for near-real-time monitoring applications. The peak GPU memory usage during inference is 34.09 MB, well within the capacity of standard edge devices. The total training time for 60 epochs is approximately 2.21 min (averaging 2.21 s per epoch), which is highly efficient for offline model development.

Among the ablation variants, removing the attention mechanism reduces FLOPs to 1.531 M (only 0.6% reduction) but increases RMSE by 24.4%, confirming that attention provides substantial accuracy gains with almost no computational overhead. Removing the LSTM module reduces FLOPs to 0.182 M (an 88.2% reduction) but increases RMSE by 30.8%, indicating that while LSTM is computationally heavier, it delivers critical temporal modeling capability. The Basic_QR model, with only 0.086 M FLOPs, achieves the lowest computational cost but suffers the worst accuracy (RMSE: 16.299). These trade-offs demonstrate that the additional computational overhead of our full architecture is justified by substantial accuracy gains, and the absolute cost remains well within practical limits for industrial deployment. This efficiency analysis, combined with the superior predictive performance reported in previous sections, supports the viability of our approach for real-world predictive maintenance systems.

## 5. Conclusions and Outlook

Aiming at the limitation of traditional time-series point prediction being unable to quantify prediction uncertainty and the shortcomings of existing probabilistic prediction methods in practicality, this paper proposes an innovative hybrid deep learning framework based on in-depth analysis of time-series data characteristics and engineering requirements. Experimental verification on the C-MAPSS dataset proves the effectiveness and superiority of this framework in RUL uncertainty quantification.

The proposed LSTM–attention–quantile regression hybrid model performs excellently in multiple dimensions. The model fully utilizes the temporal modeling capability of LSTM, the feature selection advantage of the attention mechanism, and the uncertainty quantification characteristics of quantile regression, achieving excellent performance of RMSE 11.245 and NASA Score 166.414 on the C-MAPSS dataset. Particularly noteworthy is that the model can provide an 80% prediction interval with good statistical properties, achieving a coverage rate of 86.7%, and the interval width adaptively adjusts with the RUL value, fully reflecting the uncertainty characteristics at different life stages. Ablation experiments further confirm the indispensable role of each component, providing strong support for the rationality of the model design.

We acknowledge that a limitation of the current framework is its reliance on three fixed quantiles rather than a full continuous distribution. While this design is motivated by engineering practicality—as these three levels (10%, 50%, 90%) are directly interpretable for maintenance decisions—and our results confirm that the achieved coverage (83.7%) exceeds the nominal 80% target, indicating sufficient calibration, we recognize the theoretical value of a full SQR implementation. Future work will extend this framework to Simultaneous Quantile Regression with uniformly sampled quantile levels, enabling comprehensive continuous uncertainty estimation and richer probabilistic outputs.

This research can be further explored and studied in aspects such as model optimization, technology fusion, and practical application. First, at the model level, advanced architectures such as Transformer and Graph Neural Networks can be introduced to better mine spatiotemporal correlation features in sensor data. Second, in terms of uncertainty quantification, the fusion of Bayesian deep learning and quantile regression can be explored to distinguish between epistemic uncertainty and aleatoric uncertainty. Finally, at the engineering application level, it is necessary to focus on the robustness of the model under complex working conditions, such as data missing and distribution drift, and develop corresponding online adaptive mechanisms. In addition, promoting the lightweight deployment of the model on edge devices to achieve real-time RUL prediction and decision support will be a key direction for the large-scale implementation of predictive maintenance.

Despite the promising results, the current approach has several limitations that warrant further investigation. First, while the quantile regression framework and dropout regularization provide some resilience to outliers, the model’s performance under significant sensor noise has not been systematically evaluated. Second, the model assumes complete input sequences and does not inherently handle missing sensor data, which is common in real-world industrial settings. Future work will address these limitations by incorporating robust loss functions and data augmentation techniques to improve noise resilience, integrating missing data imputation methods (e.g., interpolation or generative models), and systematically evaluating model robustness on benchmark datasets with controlled noise and missingness patterns.

## Figures and Tables

**Table 1:** Nomenclature of mathematical symbols.

Symbol	Description
*T*	Length of multivariate sensor sequence
**x** *_t_*	Feature vector at time step
*d*	Number of input features
*y*	Remaining Useful Life (RUL)
*τ*	Quantile level
*F_Y_*(⋅)	Conditional cumulative distribution function
*ρ_τ_*(*u*)	Quantile loss function (check function)
${\hat{y}}_{\tau }$	Predicted conditional quantile at level τ
*L*	Quantile regression loss
*λ*	Regularization coefficient
*L_NASA_*	Early prediction penalty term
*w*(*y*)	RUL-aware weight function
*γ*	Penalty threshold
*η*	Transition smoothness parameter
*C*	Prediction interval coverage
*W*	Normalized interval width
*α*	Weight for NASA penalty term
*β*	Weight for interval quality regularization
*s_L_*, *s_M_*, *s_U_*	Learnable scaling parameters for quantiles
*σ*(⋅)	Sigmoid function
*q*	Adjustment threshold for scaling parameters
*L_cons_*	Constraint term for scaling parameters
*W_max_*	Maximum allowed interval width
*r_target_*	Target upper/lower interval ratio
*N*	Number of samples
*L*	Sequence length (number of time steps)
*H*	Hidden dimension of LSTM
*h*	Number of attention heads

**Table 2:** FD001 dataset statistical characteristics.

Metric	Training Set	Test Set
Number of Engines	100	100
Total Time Steps	20,631	13,096
Average Life Cycles	206.31	130.96
Minimum Life Cycles	128	31
Maximum Life Cycles	362	303

**Table 3:** Hyperparameters for proposed model.

Parameter	Name	Value
Data Parameters	Sequence Length	35
	Batch Size	64
	Quantile Set	[0.1, 0.5, 0.9]
	Max RUL Value	125
Model Parameters	Hidden Dimension	48
	LSTM Layers (Bidirectional LSTM)	1
	Number of Attention Heads	8
	Feature Encoder Hidden Dim	72
	Output MLP Hidden Dims	[96, 48]
	Dropout Rate	0.22
Training Parameters	Learning Rate	8 × 10^−5^
	Optimizer	AdamW
	Weight Decay	1 × 10^−4^

**Table 4:** Performance analysis of the model in various RUL intervals.

RUL Interval	Number of Samples	RMSE	MAE	NASA Score	Coverage	Interval Width
[0, 30]	25	4.004	3.132	9.335	0.92	11.758
[30, 60]	14	8.952	8.137	16.824	0.857	24.538
[60, 90]	13	18.377	15.365	66.138	0.615	35.282
[90, 125]	37	10.122	7.907	45.904	0.943	40.918
Overall	100	11.245	8.548	166.414	0.867	30.398

**Table 5:** Comparison of performance of various models.

Model	RMSE	NASA Score
DA-LSTM [12]	12.62	263
Multi-head CNN [18]	12.17	Nan
Attn-LSTM [49]	14.53	322
AGCNN [19]	12.42	226
S-F Attention [26]	11.85	214
Liu et al. [20]	12.25	198
Res-HSA [50]	11.91	227
DAST [22]	12.88	279
AGCTE [21]	12.46	259
OURS	11.24	166

**Table 6:** Ablation experiment performance comparison.

Model	RMSE	NASA Score	Coverage	Interval Width	Parameter	FLOPs (M)
Full_Model	11.245	166.414	0.867	30.398	112,326	1.540
No_Attention	13.989	298.890	0.908	36.413	65,766	1.531
No_LSTM	14.707	440.573	0.857	41.429	34,662	0.182
No_NASA_Optimization	13.241	322.046	0.867	34.719	112,323	1.540
Basic_QR	16.299	429.608	0.827	45.134	86,723	0.086

**Table 7:** Ablation experiment performance degradation analysis.

Ablated Component	NASA Score Decrease	RMSE Decrease	Key Observation
Attention Mechanism	+79.6%	+24.4%	Significant drop in prediction accuracy, slightly better coverage but increased width
LSTM Module	+164.8%	+30.8%	Most severe performance drop; lack of temporal modeling capability has the greatest impact
NASA Optimization Module	+93.5%	+17.8%	NASA Score deteriorates significantly, engineering practicality greatly reduced
All Simplified (Basic_QR)	+158.2%	+44.9%	Worst comprehensive performance, verifies necessity of full architecture

## Data Availability

The original data presented in the study are openly available in [Kaggle] at [https://www.kaggle.com/datasets/behrad3d/nasa-cmaps]. accessed on 20 July 2026.
